# Quantifying changes in retinal non-perfusion over time with ultra-widefield fluorescein angiography following intravitreal treatment of diabetic retinopathy

**DOI:** 10.1186/s12886-026-04708-w

**Published:** 2026-03-09

**Authors:** Callie Deng, Karthik Reddy, Yue Liang, Nikhil Bommakanti, Julie Rosenthal, Yannis M. Paulus

**Affiliations:** 1 Department of Ophthalmology and Visual Sciences, W. K. Kellogg Eye Center, University of Michigan, Ann Arbor, MI USA; 2 New England Eye Center, Tufts Medical Center, Boston, MA USA; 3 Department of Ophthalmology, Wilmer Eye Institute, Johns Hopkins University, 600 N. Wolfe St, Maumenee 844A, Baltimore, MD 21287 USA; 4 Department of Biomedical Engineering, Johns Hopkins University, Baltimore, MD USA

**Keywords:** Diabetic retinopathy, Ultra-widefield fluorescein angiography, Anti-VEGF, Steroid treatment, Non-perfusion area

## Abstract

**Background:**

Diabetic Retinopathy (DR), a leading cause of preventable blindness, is conventionally evaluated using seven-field retinal fundus photography. Ultra-widefield fluorescein angiography (UWF FA) allows for detailed visualization and quantification of peripheral retinal non-perfusion (NP). This study aims to assess changes in NP and neovascularization (NV) following intravitreal anti-VEGF and steroid treatments using UWF FA.

**Methods:**

This retrospective, single-center cohort study included 65 eyes from 45 patients with Type 1 or 2 diabetes mellitus (DM) and UWF FA images acquired across two visits. Images were manually segmented by masked, trained graders, annotating foveal avascular zone (FAZ), NP, and NV surface areas. Patient demographics, clinical history, and treatment details were extracted via chart review. Stepwise multivariate linear regression identified predictors of changes in FAZ, NP, and NV areas, while causal modeling estimated effects of anti-VEGF and steroid treatments over time.

**Results:**

Eyes treated with steroid injections (*N* = 13) had decreased total and mid-periphery retinal NP areas compared to untreated eyes or eyes that received anti-VEGF alone (total change in NP area: 4.32 ± 24.66 vs. 32.30 ± 65.40 vs. 27.21 ± 42.09 mm^2^; *p* < 0.05). Increased frequency of steroid injections predicted decreased FAZ area (*p* = 0.0036). Longer duration between FAs was associated with larger NP areas by 0.87 mm^2^ per additional month (95% CI, 0.25 to 1.5; *p* = 0.0072), whereas more steroid injections (*p* = 0.035), DME on initial clinical presentation (*p* = 0.013), and vitreous hemorrhage development (*p* = 0.0002) were associated with decreased NP areas. Compared to eyes in type 1 diabetics, eyes with type 2 DM were associated with − 56.45 mm^2^ smaller total NP areas over time (95% CI, -92.3 to -20.52; *p* = 0.0028). Causal analyses suggest each additional steroid injection was associated with a 31.38 mm^2^ decrease in total NP area (95% CI, -58.14 to -4.62; *p* = 0.022). Anti-VEGF treatment (*N* = 28) did not have a significant effect on FAZ (95% CI, -0.153 to 0.060; *p* = 0.40), NP (95% CI, -17.20 to 3.91; *p* = 0.22), or NV areas (95% CI, -0.391 to 0.282; *p* = 0.75).

**Conclusions:**

Compared to anti-VEGF, intravitreal steroids produced significant improvement in retinal perfusion. Quantitative biomarkers from UWF FA may provide an objective approach to assessing treatment response and DR severity over time.

**Trial registration:**

Not applicable.

**Supplementary Information:**

The online version contains supplementary material available at 10.1186/s12886-026-04708-w.

## Background

Diabetic Retinopathy (DR) is the leading cause of preventable blindness, responsible for moderate to severe visual impairment in 3.28 million people globally in 2020 [1]. In the United States, where one-quarter of individuals with diabetes are living with DR, the prevalence is projected to grow from 9.6 million in 2021 to 16 million individuals by 2050 [2]. The primary vision-impairing complications of diabetic retinopathy include diabetic macular edema (DME) caused by leakage through the disrupted blood-retina barrier, vitreous hemorrhage, macular ischemia, and tractional retinal detachments provoked by aberrant neovascularization in proliferative diabetic retinopathy (PDR) [3].

The current gold standard for disease prognostication is seven-field retinal fundus photography, defined by the Early Treatment Diabetic Retinopathy Study (ETDRS) [4]. However, the advent of ultra-widefield (UWF) fluorescein angiography has enabled visualization and accurate quantification of previously unobserved peripheral areas of non-perfusion. A single UWF image can capture 200 degrees or 82% of the retinal surface– about two to three times more retinal surface area than the ETDRS 7-standard field protocol [5–7]. Numerous studies have demonstrated the utility of UWF imaging for improved accuracy of disease classification, detection of vision-threatening peripheral lesions, and prediction of disease progression [8–11].

From a treatment perspective, intravitreal anti-VEGF agents are first-line therapy for center-involving DME and are used as an adjunct to pan-retinal photocoagulation (PRP) for managing proliferative diabetic retinopathy [12–17]. In patients with PDR, anti-VEGF administration was associated with regression of retinal neovascularization, and when used as an adjunct to PRP, resulted in greater regression of active neovascularization than PRP alone [18–21]. Secondary analyses of the DRCR.net Protocol T and PANORAMA trials have shown eyes with nonproliferative diabetic retinopathy (NPDR) treated with bevacizumab, ranibizumab, or aflibercept have also been associated with clinically significant improvements in Diabetic Retinopathy Severity Scale (DRSS) score, with fixed dosing potentially needed to sustain improvements [14, 22, 23].

Intraocular corticosteroids including triamcinolone, dexamethasone, and fluocinolone have also been extensively studied for treatment of DME, although they are often reserved for cases unresponsive to anti-VEGF therapy, given complications associated with long-term use such as glaucoma and cataracts [14]. As potent anti-inflammatory agents, corticosteroids modulate a broad range of inflammatory mediators including cell adhesion molecules, VEGF, and other cytokines, reducing the permeability of retinal microvasculature, and inhibiting angiogenesis [24, 25]. Intravitreal dexamethasone implants developed to provide sustained drug release have also been reported to delay progression of DR [26].

While considerable work has been published on the utility of UWF FA for improved disease classification and prognostication, to our knowledge, longitudinal UWF studies that characterize the effects of DR intravitreal treatments on retinal perfusion and angiogenesis are limited. Recently, two research groups that applied UWF FA to follow DR eyes treated with anti-VEGF reported non-perfusion did not change significantly from baseline, whereas DR severity improved significantly [27, 28]. Meanwhile, a small pilot study using UWF FA to follow eyes up to one year after treatment with dexamethasone implants revealed sustained improvement in retinal perfusion [29].

The primary objective of this work is to leverage ultra-widefield imaging to quantify longitudinal changes in non-perfusion and neovascularization in the retinal periphery. Quantitative measurements of the foveal avascular zone (FAZ), non-perfusion (NP), and neovascularization (NV) areas in the retina may provide a more objective approach to characterizing disease progression and assessing intravitreal treatment response over time.

## Methods

A retrospective, comparative cohort study was conducted on patients diagnosed with type 1 or 2 diabetes mellitus who received ultra-widefield fluorescein angiography (UWF FA) at the University of Michigan W. K. Kellogg Eye Center between January 2009 and May 2018. Ethics approval was obtained from the University of Michigan Institutional Review Board (HUM00120509, PI: Y. M. Paulus) and the study adhered to the tenets of the Declaration of Helsinki. Given the retrospective nature of this study, a waiver of consent was obtained from the University of Michigan Institutional Review Board in accordance with the Health Insurance Portability and Accountability Act.

### Inclusion and exclusion criteria

**Fig. 1:**
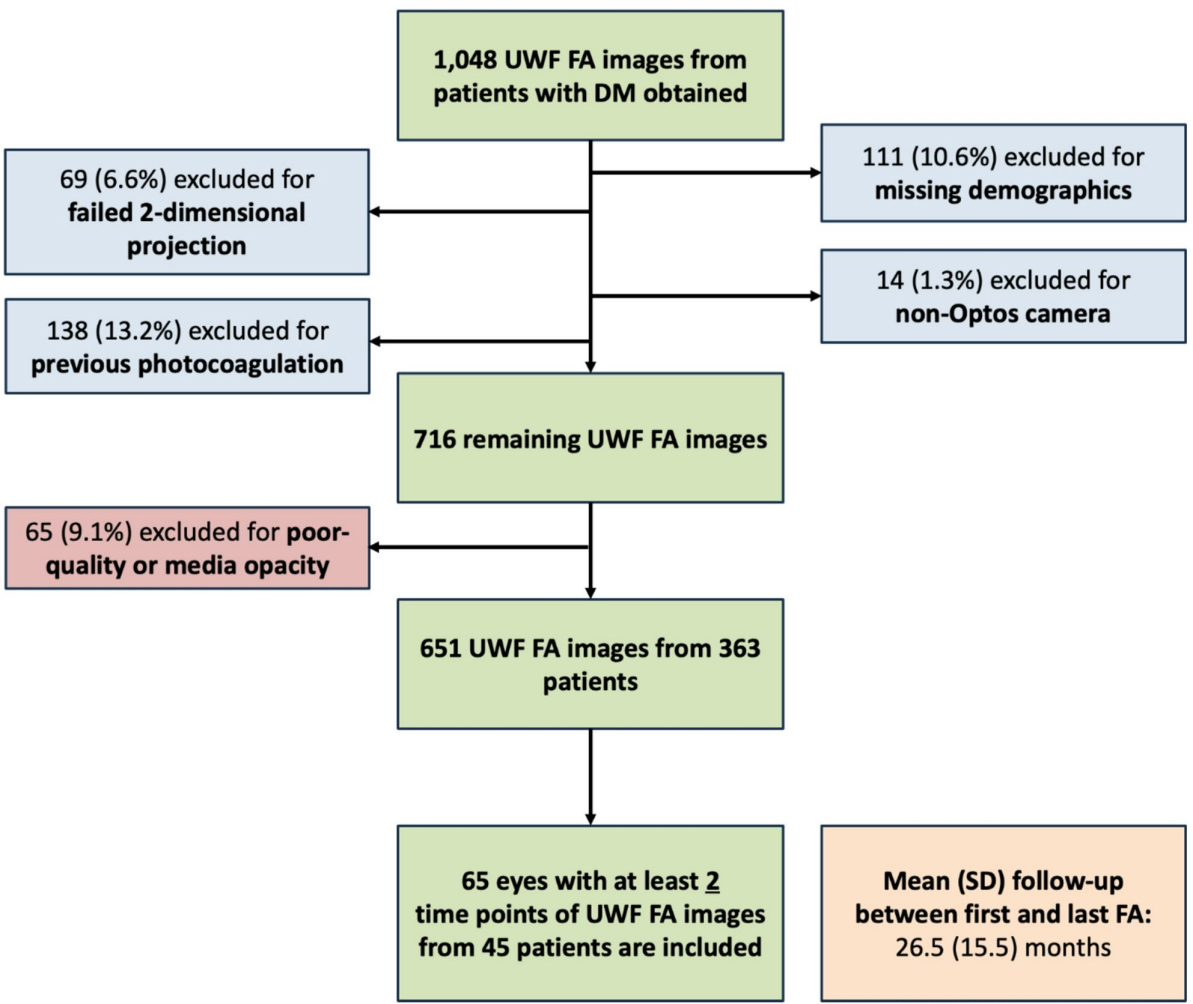
Flow diagram showing initial and final patient cohort size, following exclusion based on exclusion criteria

Eligible participants met the following inclusion criteria: age of 18 years or older, a diagnosis of type 1 or 2 diabetes mellitus, two or more examinations using dilated fundus examination and UWF FA at Ann Arbor, Michigan, or the Grand Blanc, MI, satellite clinic. Exclusion criteria were as follows: evidence of prior treatment with pan-retinal photocoagulation, poor image quality resulting in indistinguishable areas of NV and NP, or abundant media opacities such as significant vitreous hemorrhage or cataracts that obscured visualization of the retina on UWF FA (Fig. 1). Patients who had received prior intravitreal anti-VEGF or steroid treatments were not excluded.

### Ultra-widefield FA and image segmentation

Arteriovenous-phase FA images were obtained approximately 20–45 s post-injection using Optos 200Tx or California (Optos PLC, Scotland, UK) scanning laser ophthalmoscopes. Specifically, the first, clearest frame in this period demonstrating laminar venous filling with minimal leakage was included, with use of the nearest adjacent frame, as needed, in the case of poor image quality or opacities. Images were projected onto a curved surface with a nominal eye diameter of 24 mm using previously described proprietary Optos software [30].

**Fig. 2:**
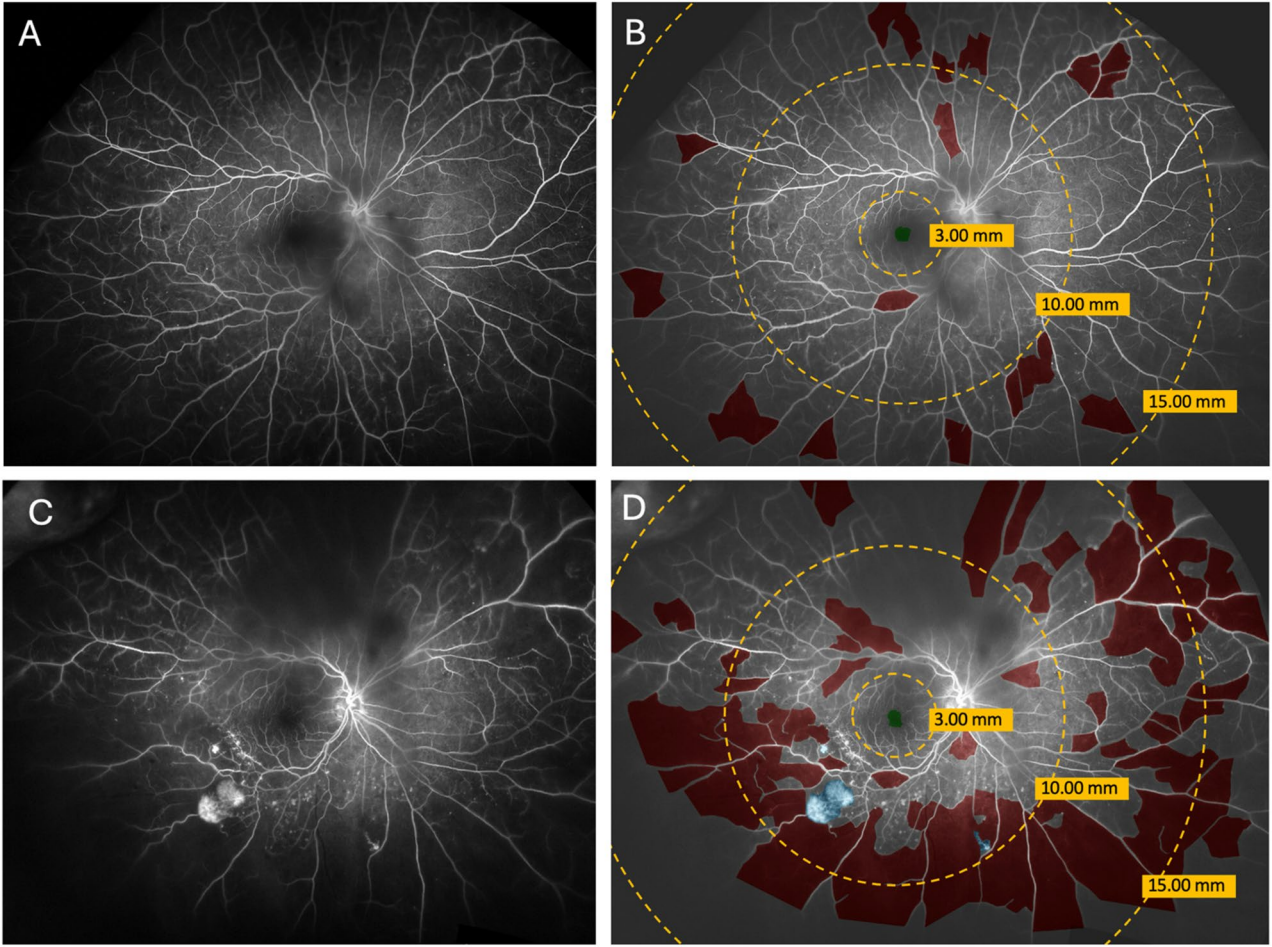
Example ultra-widefield fluorescein angiogram images acquired over time. (**A**) Example of a baseline ultra-widefield FA image and (**C**) follow-up FA of the same eye, acquired 21 months apart. (**B**,** D**) UWF FA images overlayed with segmentations of areas of non-perfusion (red), neovascularization (blue), and foveal avascular zone (green). Areas were totaled for each region: the posterior pole included within a 3.00 mm radius of the identified FAZ, the mid periphery included between a 3.00 and 10.00 mm radius, and the far periphery included between10.00 and 15.00 mm radius of the FAZ

All image segmentations were completed using Insight Tool- kit SNAP (ITK-SNAP), an open-source application that allows level set active contour segmentation of 3-dimensional images (University of North Carolina Chapel Hill, Insight Toolkit) [31]. Each image was manually segmented by one of four masked and trained graders, annotating FAZ, NP, and NV surface areas (Fig. 2).

NP and NV surface areas were subdivided into posterior pole, mid periphery, and far periphery regions based on distance from the FAZ. The posterior pole was defined as the region within a 3.00 mm radius or less of the identified FAZ; the mid periphery included beyond 3.00 mm and up to 10.00 mm; and the far periphery included beyond 10.00 mm up to 15.00 mm. FAZ, NP, and NV surface areas were calculated for each image in square millimeters, and changes in surface areas were calculated by subtracting areas of the first scan from the most recent scan. Further detailed descriptions of the above methodology can be found in our prior manuscript [32].

### Statistical analysis

All statistical analyses were performed using the R programming language (version 4.3.3) [33]. Both parametric and nonparametric statistics were used in the statistical comparisons. Preliminary univariate analyses were conducted to compare patient demographics and changes in surface areas between treated and untreated eyes, using independent samples t-tests for continuous outcomes. To account for repeated measures within patients and the correlation between eyes, linear mixed models were fitted using the *lme4* package for continuous variables such as changes in FAZ, NP, and NV surface areas. The models included fixed effects for treatment type, initial clinical severity, and patient demographic factors with the goal of evaluating the contribution of each of these factors to changes in FAZ, NP, and NV surface areas over time. Statistical significance was defined as a p-value less than 0.05.

Stepwise multivariate linear regression was performed using the *car* package to identify factors that were statistically significant predictors in changes in FAZ, NP, and NV areas over time. All available demographic factors, clinical patient factors, and treatment factors were included in the initial model. Demographic factors included sex, age, race, and type of diabetes mellitus; clinical patient factors included last hemoglobin a1c, initial severity of DR on presentation by clinical exam, initial DME on presentation by clinical exam, development of DME, development of VH, and time elapsed between FAs to account for natural disease progression; treatment factors included whether or not the patient received anti-VEGF or steroid treatment and the number of injections they receive between FAs. Covariates were individually dropped from the model, starting with the least significant based on p-values to ultimately identify the most parsimonious model. Interaction terms were also added to the linear mixed models to evaluate the effect modification by clinical severity and treatment type, specifically exploring the interactions between diabetic retinopathy severity and anti-VEGF treatment as well as between diabetic macular edema and anti-VEGF treatment. The ETDRS classification system was used to determine clinical severity, with no DR, mild NPDR, moderate NPDR, severe NPDR, or PDR corresponding to an ETDRS score of 0–4, respectively.

To estimate the causal effects of anti-VEGF and steroid treatments on changes in FAZ, NP, and NV, inverse propensity weighting was applied using the *twang* package. Propensity scores were calculated using logistic regression for binary variables and generalized linear propensity scores for continuous variables. The outcome model included the weighted outcomes of interest, with treatment variables incorporated in a saturated model: Outcome ~ treatments. This approach mitigated confounding by balancing the distribution of covariates across treatment groups.

## Results

The initial dataset, previously described by Yu et al., comprised 651 eyes from 363 patients with type 1 or 2 diabetes mellitus [32, 34]. A subset of 65 eyes from 45 patients had UWF FA images taken across at least two time points for analysis in this study. For this cohort, the mean (SD) patient age was 58.8 (12.9) years, and 37.8% (17) of patients were female. In total, 73.3% (33) of patients self-identified as white, 17.8% (8) as non-Hispanic black, 4.4% (2) as Asian, and 4.4% (2) as Hispanic other. Type 2 diabetes was present in 84.4% (38) of patients included (Table 1). Of the 65 eyes, 3 eyes initially presented with no DR, 6 eyes with mild NPDR, 18 eyes with moderate NPDR, 13 eyes with severe NPDR, 14 eyes with PDR, and 14 eyes with unknown severity. The mean (SD) time elapsed between the first and last FA was 26.5 (15.5) months. A total of 28 eyes with UWF FA images acquired across two visits had received intravitreal anti-VEGF treatment with a mean (range) of 4.7 (1–22) anti-VEGF injections per eye. Thirteen eyes received steroid treatment and had more than 1 fluorescein angiogram, with a mean (range) of 2.3 (1–6) injections per eye.

**Table 1:** Patient and eye-level characteristics

Variable	No. (%)
Patients	45
Age, mean (SD)	58.8 (12.9)
Sex	
Male	28 (62.2)
Female	17 (37.8)
Race/ethnicity	
White	33 (73.3)
Non-Hispanic Black	8 (17.8)
Asian	2 (4.4)
Hispanic Other	2 (4.4)
Diabetes Mellitus	
Type 1	7 (15.6)
Type 2	38 (84.4)
Eyes	65
Severity of DR on Initial Presentation	
None	3 (4.6)
Mild NPDR	6 (9.2)
Moderate NPDR	18 (27.7)
Severe NPDR	13 (20.0)
PDR	11 (16.9)
Unknown	14 (21.5)
Severity of DR on Final Presentation	
None	0 (0)
Mild NPDR	6 (9.2)
Moderate NPDR	17 (26.2)
Severe NPDR	15 (23.1)
PDR	21 (32.3)
Unknown	6 (9.2)
Developed DME	
Yes	49 (75.4)
No	16 (24.6)
Developed Vitreous Hemorrhage	
Yes	4 (6.2)
No	61 (93.8)
Type of Treatment Received	
No Treatment	35 (53.8)
Anti-VEGF	28 (43.1)
Mean Injections between FA images (range)	4.7 (1–22)
Steroids	13 (20.0)
Mean Injections between FA images (range)	2.3 (1–6)

Thirty-five out of 65 eyes (53.8%) did not receive any treatment during the study, whereas 17 eyes (26.2%) were treated with anti-VEGF only. Of the remaining 13 eyes that received steroid injections (20.0%), 11 had also been treated with anti-VEGF. The number of intravitreal treatments for each patient and eye are shown in Supplemental Table 1.

Initial univariate analysis suggested no statistically significant difference in changes in FAZ, non-perfusion, and neovascularization areas between untreated eyes and those treated with anti-VEGF only (Tables 2 and 3). On the other hand, eyes treated with steroid injection had decreased total NP area compared to those untreated (4.32 ± 24.66 mm^2^ vs. 32.30 ± 65.40 mm^2^, *p* = 0.018) and eyes treated with anti-VEGF only (4.32 ± 24.66 mm^2^ vs. 27.21 ± 42.09 mm^2^, *p* = 0.037). Differences between eyes treated with steroids and those that received no treatment were also statistically significant for mid-periphery and far-periphery NP areas (Table 3). Given the sparsity of NV data in a relatively small sample size, statistical significance could not be determined for changes in NV with treatment.

**Table 2:** Comparison of patient characteristics and UWF FA biomarkers across treatment subgroups

**Variable**	No Treatment		Anti-VEGF Only		Steroid +/- Anti-VEGF	
	*(* *n* * = 35)*		*(n = 17)*		*(n = 13)*	
	**Mean**	**(SD)**	**Mean**	**(SD)**	**Mean**	**(SD)**
Initial Age	54.54	(14.26)	58.52	(13.15)	65.43	(7.93)
Years with DM (rounded)	16.17	(9.15)	15.00	(5.74)	20.54	(9.50)
Months Elapsed Between FAs	23.11	(13.31)	29.03	(15.16)	34.27	(20.15)
Delta FAZ Area in mm^2^	0.05	(0.59)	0.13	(0.40)	-0.13	(0.44)
Delta Total NP Area in mm^2^	32.30	(65.40)	27.21	(42.09)	4.32	(24.66)
Delta NP Posterior Pole Area	0.82	(3.61)	0.85	(2.98)	0.11	(0.28)
Delta NP Mid-Periphery Area	9.30	(22.77)	9.40	(20.31)	-0.51	(5.32)
Delta NP Far-Periphery Area	22.17	(45.14)	16.96	(37.97)	4.72	(21.18)
Delta Total NV area in mm^2^	0.55	(1.60)	0.41	(1.66)	-0.02	(0.07)

Delta FAZ area - Change in Foveal Avascular Zone area; Delta Total NP area – Change in Total Non-perfusion area; Delta Total NV area – Change in Total Neovascularization area

**Table 3:** Univariate statistical analyses comparing UWF FA biomarkers across treatment subgroups

Variable	*P*-Value		
	No Treatment vs. Anti-VEGF Only	Anti-VEGF Only vs. Steroid	No Treatment vs. Steroid
Delta FAZ Area in mm^2^	0.298	0.054	0.124
Delta Total NP Area in mm^2^	0.368	0.037*	0.018**
Delta NP Posterior Pole Area	0.487	0.161	0.128
Delta NP Mid-Periphery Area	0.494	0.035*	0.011**
Delta NP Far-Periphery Area	0.333	0.136	0.038*
Delta Total NV area in mm^2^	*Omitted from analysis due to data sparsity.*		

Delta FAZ area - Change in Foveal Avascular Zone area; Delta Total NP area – Change in Total Non-perfusion area; Delta Total NV area – Change in Total Neovascularization area

* Statistically significant p-value < 0.05, ** p-value < 0.025 using Welch’s t-test

Table 4 summarizes the statistically significant predictors of change in quantitative FA biomarkers over time, based on multivariate linear regression analysis. Demographic factors including age, sex, and race were not statistically significant predictors of change in FAZ or NP area over time. Relative to type 1 DM, type 2 DM was associated with a 56.45 mm^2^ decrease in total NP area (95% CI, -92.30 to -20.52; *p* = 0.0028) over time. Additionally, the presence of DME on initial clinical presentation and development of vitreous hemorrhage previously absent on initial presentation was associated with decreased NP areas over time. Longer duration between FAs was predictive of increased NP area by 0.87 mm^2^ per month (95% CI, 0.25 to 1.50; *p* = 0.0072), equivalent to 10.44 mm^2^ per year.

A higher frequency of steroid treatment was predictive of decreased FAZ area over time (*p* = 0.0036). Each additional steroid injection was also associated with an 11.96 mm^2^ decrease in NP area (95% CI, -22.95 to -0.86; *p* = 0.0349). Conversely, the quantity and frequency of anti-VEGF treatment were not predictive of changes in FAZ or NP areas over time. Older patient age may be associated with decreased NV area over time, and the development of vitreous hemorrhage not seen on initial presentation may be associated with increased NV area over time. However, conclusions about statistical significance cannot be drawn, given the limited sample size.

**Table 4:** Statistically significant predictors of changes in FAZ, total NP, and NV areas over time

Biomarker	Variable	Coefficient (mm^2^)	Standard Error	95% CI	*p*-value
Delta FAZ	Steroid treatment frequency (injections per month)	-4.012	1.259	-6.54, -1.50	0.0036**
Delta Total NP	Type 2 vs. 1 diabetes mellitus	-56.446	17.961	-92.30, -20.52	0.0028**
	DME on initial clinical exam	-28.367	11.078	-50.59, -6.20	0.0128*
	Developed vitreous hemorrhage	-77.270	19.596	-116.26, -37.97	0.0002***
	# of steroid injections	-11.956	5.524	-22.95, -0.86	0.0349*
	Time elapsed (months)	0.874	0.314	0.25, 1.50	0.0072**
Delta Total NV	Initial Age	-0.038	0.012	-0.06, -0.01	0.0020**
	Developed vitreous hemorrhage	1.618	0.628	0.37, 2.87	0.0123*

Delta FAZ area - Change in Foveal Avascular Zone area; Delta Total NP area – Change in Total Non-perfusion area; Delta Total NV area – Change in Total Neovascularization area

Statistical significance denotated as * *p* < 0.05, ** *p* < 0.01, *** *p* < 0.001

Table 5 reports the estimated causal contributions of treatment with anti-VEGF and steroid injections to changes in retinal areas over time. The causal model suggests that anti-VEGF treatment had no statistically significant effect on FAZ, total NP, and total NV areas. Similarly, the effect of steroid treatment on the FAZ area was not significant. By contrast, for every additional steroid injection, the total NP area decreased by 31.38 mm^2^ (95% CI, -58.14, -4.62; *p* = 0.022). Steroid injections may also cause shrinking areas of NV over time, though the statistical significance requires verification with a larger sample size.

**Table 5:** Inferred causal effect of anti-VEGF and steroid treatment on FAZ, total NP, and NV areas

Biomarker, Mean (SD)	Variable	Coefficient (mm^2^)	Standard Error	95% CI	*p*-value
Delta FAZ 0.036 (0.521)	Treated with anti-VEGF vs. not	-0.017	0.122	-0.255, 0.222	0.892
	# of anti-VEGF injections	-0.046	0.054	-0.153, 0.060	0.396
	Treated with steroid vs. not	-0.306	0.215	-0.729, 0.116	0.155
	# of steroid injections	-0.365	0.254	-0.862, 0.133	0.151
Delta Total NP 25.370 (54.280)	Treated with anti-VEGF vs. not	3.913	15.079	-25.64, 33.47	0.795
	# of anti-VEGF injections	-6.646	5.386	-17.20, 3.91	0.217
	Treated with steroid vs. not	-24.304	12.198	-48.21, -0.397	0.046*
	# of steroid injections	-31.377	13.653	-58.14, -4.62	0.022*
Delta Total NV 0.400 (1.450)	Treated with anti-VEGF vs. not	-0.228	0.276	-0.770, 0.313	0.409
	# of anti-VEGF injections	-0.055	0.172	-0.391, 0.282	0.751
	Treated with steroid vs. not	-0.482	0.205	-0.884, -0.080	0.019*
	# of steroid injections	-0.516	0.221	-0.948, -0.083	0.019*

Delta FAZ area - Change in Foveal Avascular Zone area; Delta Total NP area – Change in Total Non-perfusion area; Delta Total NV area – Change in Total Neovascularization area

* Statistical significance denotated as p < 0.05

## Discussion

Using UWF FA images acquired over time, this study quantifies the contribution of demographic factors, clinical factors, and intravitreal treatments to changes in non-perfusion and neovascularization for eyes with DR. Our results suggest that intravitreal steroid injections significantly reduce the expansion of total and mid-peripheral retinal areas of nonperfusion over time, when compared to no treatment or anti-VEGF alone. Using inverse propensity weighting to mitigate confounding and infer the causal effect of anti-VEGF and steroid treatment, we found that intravitreal anti-VEGF did not have a significant effect on change in NP area over time. This finding supports prior studies that similarly reported non-significant changes in NP from baseline despite significant improvement in DR severity with anti-VEGF treatment [27, 28]. Meanwhile, steroid treatment had a pronounced effect, reducing total NP area by approximately 31.38 mm^2^ with each injection. This is in line with the results of a small pilot study of six eyes treated with intravitreal dexamethasone implants, where UWF FA imaging confirmed sustained reductions in peripheral non-perfusion [29].

Potential mechanisms for this phenomenon could include steroid-related suppression of inflammatory and VEGF pathways, allowing attenuation of underlying ischemic and neovascular processes. In vivo models have demonstrated steroid-mediated reduction in VEGF and ICAM-1 expression with upregulation of FLT-1 in retinal vasculature that may underlie this process [25, 35], however further work must be done to ascertain why anti-VEGF has uncertain efficacy in attenuation of non-perfusion [36]. Regardless, further larger prospective studies must be performed to better gauge and more definitively compare treatment effects between treatment types.

We found that demographic factors, including age, sex, and race, do not have a significant effect on changes in the FAZ or NP areas longitudinally over time. Previous manuscripts investigating the impact of demographics on total retinal non-perfusion and neovascularization using a cross-sectional approach with a single time point by Yu et al. reported that male sex and black race were strongly associated with greater areas of non-perfusion and neovascularization [32].

By contrast, the time elapsed time between FAs, which can be interpreted as a surrogate for the natural progression of the disease, was predictive of an increase in total NP area by 10.44 mm^2^ per year. These results agree with a prior cross-sectional study that assessed changes in NP using “time since diagnosis” as a proxy for disease progression and inferred a relationship between each additional year of age with an increase in NP area by 10.75 mm^2^ [34]. Using the previously reported threshold of 77.48 mm² of total NP area for increased neovascularization risk, our results indicate an average of 7.4 years of progressive retinal non-perfusion to reach the threshold for PDR [32].

Our results also suggest eyes of patients with type 2 DM may have attenuated changes in NP area over time compared to those with type 1 DM. Eyes of patients with type 2 DM may appear to have less non-perfusion because of the later onset of pathology and different treatment exposures. Other reports have demonstrated a trend of greater proportion of type 1 DM in cohorts with higher levels of non-perfusion on baseline imaging [37]. Additionally, type 1 DM may be correlated with higher DR severity scores than type 2 DM and may be associated with larger areas of non-perfusion [38–41]. Given the limited sample size, these findings warrant further investigation across larger, prospective cohorts to make definitive conclusions on the relationship between diabetes type and retinal non-perfusion. Finally, development of vitreous hemorrhage was also predictive of decreased areas of retinal non-perfusion. A potential explanation is that the presence of vitreous hemorrhage may mask areas of capillary dropout and reduce grader accuracy in detecting non-perfusion areas.

A major limitation of this study is the small sample size of eyes with at least 2 UWF fluorescein angiography images, which reduced the power and statistical significance of our results. To mitigate possible confounding, such as the natural history of the disease and the number of anti-VEGF or steroid injections received, multivariate regression and causal analyses were performed. From a methodology perspective, we calculated areas using a fixed 24-mm globe model, as previously reported in the literature, which may shift absolute surface measurements in eyes with atypical axial lengths [30, 32]. Additionally, image quality and inter-grader variability may affect quantification of NP and NV areas. While a senior retinal specialist reviewed measurements for consistency and adjudicated equivocal cases, we did not perform formal repeatability testing, and therefore results should be interpreted with caution. Future work will aim to incorporate biometric axial length to enable subject-specific scaling and repeatability studies to ensure improved accuracy and consistency of measurements. Lastly, our study was limited to injections administered after the patient began care within our eye center. Intravitreal injections performed prior to care at our facility could lead to incomplete treatment counts and limit the accuracy of our findings. Further investigation with a larger, prospective study is warranted.

## Conclusion

This longitudinal ultra-widefield FA study illustrates how quantitative mapping of peripheral retinal pathology can track disease progression and treatment response in diabetic retinopathy. Across serial imaging, intravitreal steroid, and not anti-VEGF treatment alone, was associated with significant attenuation of nonperfusion area. Demographic factors did not significantly influence longitudinal changes in FAZ or NP. These findings, taken together, support the value of UWF-based quantification for objective monitoring and suggest steroids may play a distinct role in mitigating peripheral ischemia.

While interpretation may be limited by the study’s sample size and retrospective design, the findings of this pilot study can inform future prospective studies that utilize standardized treatment protocols to more rigorously characterize and validate treatment response. By quantifying the effects of demographics, clinical presentation, and treatment with intravitreal anti-VEGF and steroids, our study enhances the utility and interpretation of quantitative biomarkers, such as non-perfusion area, for assessing DR severity and treatment response.

## Supplementary Information

Below is the link to the electronic supplementary material.

Supplementary Material 1

## Data Availability

The de-identified, anonymized datasets generated and/or analyzed during the current study are available from the corresponding author on reasonable request.

## Abbreviations

DR: Diabetic retinopathy
UWF FA: Ultra-widefield fluorescein angiography
FAZ: Foveal avascular zone
NP: Non-perfusion
NV: Neovascularization
DM: Diabetes mellitus
DME: Diabetic macular edema
PDR: Proliferative diabetic retinopathy
ETDRS: Early Treatment Diabetic Retinopathy Study
PRP: Pan retinal photocoagulation
NPDR: Nonproliferative diabetic retinopathy
DRSS: Diabetic Retinopathy Severity Scale
SD: Standard deviation
