# Supplementary material for: Quantifying changes in retinal non-perfusion over time with ultra-widefield fluorescein angiography following intravitreal treatment of diabetic retinopathy
Source: BMC Ophthalmol. 2026 Mar 9;26:183. doi: 10.1186/s12886-026-04708-w (PMC13085603; doi:10.1186/s12886-026-04708-w)
Supplement: Supplementary file 1 — Supplementary Material 1 [file 12886_2026_4708_MOESM1_ESM.docx]

**Supplemental Table 1.** Number of treatments per patient and eye included in our study.

| Patient ID | Laterality | # of Anti-VEGF Injections between FAs | # of Steroid Injections between FAs |
| --- | --- | --- | --- |
| 1 | OS | 0 | 0 |
| 2 | OD | 1 | 0 |
| 2 | OS | 0 | 0 |
| 3 | OD | 0 | 0 |
| 4 | OD | 3 | 0 |
| 4 | OS | 3 | 0 |
| 5 | OD | 5 | 5 |
| 5 | OS | 5 | 6 |
| 6 | OD | 0 | 0 |
| 6 | OS | 0 | 0 |
| 7 | OS | 0 | 0 |
| 8 | OD | 0 | 0 |
| 8 | OS | 0 | 0 |
| 9 | OD | 0 | 0 |
| 9 | OS | 0 | 0 |
| 10 | OD | 0 | 0 |
| 11 | OD | 0 | 0 |
| 11 | OS | 0 | 0 |
| 12 | OD | 0 | 0 |
| 13 | OD | 0 | 0 |
| 14 | OS | 7 | 0 |
| 15 | OD | 0 | 0 |
| 16 | OD | 0 | 0 |
| 16 | OS | 0 | 0 |
| 17 | OS | 0 | 0 |
| 17 | OD | 0 | 0 |
| 18 | OD | 2 | 0 |
| 19 | OS | 0 | 1 |
| 20 | OD | 9 | 0 |
| 20 | OS | 5 | 0 |
| 21 | OD | 0 | 0 |
| 22 | OD | 3 | 0 |
| 22 | OS | 0 | 0 |
| 23 | OD | 0 | 0 |
| 24 | OD | 0 | 0 |
| 24 | OS | 0 | 0 |
| 25 | OD | 0 | 0 |
| 25 | OS | 0 | 0 |
| 26 | OD | 0 | 0 |
| 26 | OS | 0 | 0 |
| 27 | OD | 0 | 0 |
| 28 | OD | 2 | 0 |
| 29 | OD | 1 | 1 |
| 30 | OD | 4 | 0 |
| 31 | OD | 9 | 0 |
| 31 | OS | 10 | 0 |
| 32 | OD | 8 | 2 |
| 33 | OS | 7 | 4 |
| 34 | OS | 22 | 2 |
| 35 | OD | 0 | 0 |
| 35 | OS | 0 | 0 |
| 36 | OD | 4 | 1 |
| 37 | OS | 0 | 0 |
| 38 | OD | 2 | 0 |
| 38 | OS | 1 | 0 |
| 39 | OD | 2 | 0 |
| 40 | OS | 2 | 0 |
| 41 | OD | 1 | 1 |
| 42 | OD | 0 | 0 |
| 42 | OS | 0 | 0 |
| 43 | OD | 0 | 2 |
| 44 | OS | 2 | 1 |
| 44 | OD | 1 | 0 |
| 45 | OD | 6 | 2 |
| 45 | OS | 4 | 2 |
